# Reduction of NgR in perforant path decreases amyloid-β peptide production and ameliorates synaptic and cognitive deficits in APP/PS1 mice

**DOI:** 10.1186/s13195-020-00616-3

**Published:** 2020-04-24

**Authors:** Rong Jiang, Xue-Fei Wu, Bin Wang, Rong-Xiao Guan, Lang-Man Lv, Ai-Ping Li, Lei Lei, Ye Ma, Na Li, Qi-Fa Li, Quan-Hong Ma, Jie Zhao, Shao Li

**Affiliations:** 1 Liaoning Provincial Key Laboratory of Cerebral Diseases, Department of Physiology, College of Basic Medical Sciences, Dalian Medical University, Dalian, China; 2 National-Local Joint Engineering Research Center for Drug Research and Development (R&D) of Neurodegenerative Diseases, Dalian Medical University, Dalian, China; 3 Jiangsu Key Laboratory of Neuropsychiatric Diseases, Institute of Neuroscience, Soochow University, Suzhou, China

**Keywords:** Alzheimer’s disease, Nogo receptor, Perforant path, Amyloid precursor protein (APP), Amyloid beta, Rho/ROCK signaling pathway

## Abstract

**Background:**

Amyloid beta (Aβ) which is recognized as a main feature of Alzheimer’s disease (AD) has been proposed to “spread” through anatomically and functionally connected brain regions. The entorhinal cortex and perforant path are the earliest affected brain regions in AD. The perforant path is the most vulnerable circuit in the cortex with respect to both aging and AD. Previous data show that the origins and terminations of the perforant path are susceptible to amyloid deposition at the younger age in AD. Nogo receptor (NgR) plays an essential role in limiting injury-induced axonal growth and experience-dependent plasticity in the adult brain. It has been suggested that NgR is involved in AD pathological features, but the results have been conflicting and the detailed mechanism needs further investigation. In this study, the effect of NgR in the perforant path on the pathological and functional phenotype of APP/PS1 transgenic mice was studied.

**Methods:**

To genetically manipulate NgR expression, adeno-associated virus (AAV) with short hairpin (shRNA) against NgR was injected into the perforant path of APP/PS1 transgenic mice, followed by an assessment of behavioral, synaptic plasticity and neuropathological phenotypes. NgR was overexpressed or knockdown in neuroblastoma N2a cells and APPswe/HEK293 cells to investigate the interaction between NgR and amyloid precursor protein (APP).

**Results:**

It is shown that reduction of NgR in the perforant path rescued cognitive and synaptic deficits in APP/PS1 transgenic mice. Concurrently, Aβ production in the perforant path and levels of soluble Aβ and amyloid plaques in the hippocampus were significantly decreased. There was a positive correlation between the total APP protein level and NgR expression both in transgenic mice and in cultured cells, where the α-secretase and β-secretase cleavage products both changed with APP level in parallel. Finally, NgR might inhibit APP degradation through lysosome by Rho/Rho-associated protein kinases (ROCK) signaling pathway.

**Conclusions:**

Our findings demonstrate that perforant path NgR plays an important role in regulating APP/Aβ level and cognitive functions in AD transgenic mice, which might be related to the suppression of APP degradation by NgR. Our study suggests that NgR in the perforant path could be a potential target for modulating AD progression.

## Background

Alzheimer disease (AD) is the most common cause of dementia. AD pathology is characterized by amyloid plaque formation and neurofibrillary tangle deposition. The amyloid beta (Aβ) peptide is the derivative of amyloid precursor protein (APP) generated through sequential proteolytic processing by β- and γ-secretases. APP is a transmembrane protein whose physiological function has not yet to be fully elucidated. APP subcellular localization affects the formation of Aβ plaque [1–3]. For instance, accelerating the transfer of APP from early to late endosomes decreases the occurrence of APP β-cleavage [3]. Accumulative evidences have shown the crucial role of APP in triggering a complex pathophysiological cascade which leads to the neurodegenerative conditions observed in AD.

In AD patients, the entorhinal cortex (EC) and perforant path are the earliest affected brain regions [4, 5] in which neurofibrillary tangles and synaptic loss have been found [6–8]. In addition, animal studies have proved that modulation of neuronal activity via the perforant path could affect interstitial fluid Aβ level and possibly subsequent Aβ deposition as well [5, 9]. Intriguingly, our previous study found firstly that APP is specifically located at the node of Ranvier (NOR) in the nerve fibers [10], implicating a potential role for NORs in the Aβ release. Taken together, these studies have suggested that the perforant path might play an important role in AD and interfere Aβ metabolism in this region which may affect the progression of AD.

The Nogo-66 receptor (NgR, also termed NgR1) is an important molecule at NORs, which participates in limiting injury-induced axonal growth and experience-dependent plasticity in the adult brain [11–13]. The three myelin ligands for NgR are Nogo (also known as reticulon 4) [14–16], myelin-associated glycoprotein (MAG) [17], and oligodendrocyte myelin glycoprotein (OMgp) [18]. Most recently, intriguing studies have implicated the involvement of NgR in the pathogenesis of AD. For example, expressions of NgR are increased in patients with AD [19]. Deleting Nogo ameliorates learning and memory deficits of APP transgenic mice at an early/intermediate stage of the disease [20]. And suppression of the Nogo/NgR pathway by NEP1-40 attenuates the deposition of Aβ plaque [21]. These studies indicate that Nogo/NgR pathway might promote AD pathology. However, others have presented conflicting results. It has been reported that the absence of NgR increases Aβ plaque load in the brain of APPswe/PSEN-1ΔE9 transgenic mice [22] and subcutaneous treatment of NgR (310) ecto-Fc reduces brain Aβ plaque load and increases the relative levels of serum Aβ in the transgenic mice [23]. The controversial results might be explained by the different methods targeting NgR or Nogo. We speculate that NgR might play distinct roles in axonal pathways, and whether NgR in the perforant path regulates Aβ generation and cognitive functions in AD is of interest.

In this study, we find that reduction of NgR in the perforant path ameliorates synaptic and cognitive deficits and reduces Aβ deposition in APP/PS1 transgenic mice, which may be related to enhanced APP degradation through lysosome by Rho/Rho-associated protein kinases (ROCK) signaling pathway.

## Methods

### Animals and habituation

The male APP/PS1 transgenic mice at 6 months of age were housed in pairs, in standard cages, with free access to food and water under a 12-h light/dark cycle, controlled temperature, and humidity in specific pathogen-free conditions at the University Laboratory Animal Center.

### Plasmid construction and stereotaxic delivery

We used adeno-associated virus (AAV) vectors prepared by Obio Technology (Shanghai, China) to suppress NgR expression. The small hairpin RNA (shRNA) expression was constructed into pAKD using Bgl II and Sal I sites. For shRNA against mouse NgR, the optimal target sequence (GATCCCCGCCGAAATCTCACTATCCTTTCAAGAGA AGGATAGTGAGATTTCGGCTTTTT) was selected, and a scrambled shRNA (GATCCCCTTCTCCGAACGTGTCACGTTTCAAGAGAACGTGACACGTTCGGAGAATTTTTTGTAC) served as a control. The NgR shRNA sequence and control shRNA sequence were cloned into the pAKD-CMV-bGlobin-eGFP-H1-shRNA vector. We then used a total of 10^12^ vg/mL of the virus to transfect the perforant path regions of mice. The recombinant vectors (AAV-vector and AAV-shNgR) contained an enhanced green fluorescence protein (eGFP) as a marker to track AAV-mediated target gene expression by fluorescence microscopy. Mice were anesthetized with isoflurane and mounted onto a stereotaxic frame. AAV was injected bilaterally into the perforant path (coordinates, bregma: AP − 4.24 mm, ML ± 2.9 mm, and DV − 1.6 mm) with a slow injection rate through a microinjector attached to a digital stereotaxic arm. After injection was completed, the injector was left in place for an additional 5 min. Mice were recovered on a heating pad upon revival.

### Morris water maze assessment

The Morris water maze is used to evaluate spatial learning and memory ability of rodents [24, 25]. 8.5-month-old APP/PS1 transgenic mice were trained in the Morris water maze containing water with temperatures of 23 ± 0.5 °C. The goal platform with a diameter of 10 cm was kept submerged 1 cm below the water level. The acquisition training was performed for five successive days, and each mouse participated in 4 trials per day. For the first day, mice were allowed to find the visible platform for 90 s. From the 2nd day till the 5th day, mice were allowed to find the hidden platform. Once the mouse found the platform, it was allowed to remain there for 15 s. If the mouse failed to find the platform in 90 s, then the mouse was gently guided to the platform and allowed to remain there for 10 s. The escape latency and swimming speed were analyzed. In the test phase, the platform was removed and each mouse was allowed to swim in the pool for 90 s. Platform crossings, swimming paths, and swimming speed were recorded. Mouse activity in the aforementioned behavioral apparatuses was collected by a digital video camera connected to a computer-controlled system (Ethovision 2.0, Noldus, Wageningen, Netherlands). All tests were each blind to the treatment schedule.

### Passive avoidance test

The shuttle-box apparatus is also used to evaluate the learning and memory ability of rodents [26]. The apparatus consists of a box with an illuminated area and a dark area (each area 30 × 25 × 18.5 cm); both were equipped with a grid floor composed of steel bars. The passive avoidance task consisted of 2 trials, acquisition trial and retention trial. On the first day of training, mice were placed individually into the light compartment by a bright bulb and allowed mice to explore the boxes. The intercompartment door was opened after a 300-s acclimation period. To later adapt, if the mouse stepped into the dark compartment, an inescapable foot shock (0.1 mA/s) was delivered through the grid floor. The retention trial started 24 h after the end of the acquisition trial. Each mouse was again placed in the illuminated compartment, and the latency of mouse re-enter the dark compartment was recorded up to 300 s. The latency in the retention trial was used as the index of retention of the learning experience. No shock was applied during the retention trial.

### Y-maze spontaneous alternation test

Spontaneous alternation behavior in the Y-maze test, a behavioral test based on the animals’ natural curiosity for exploration, is considered to reflect the short-term spatial working memory [27, 28]. The apparatus for Y-maze testing is made of three opaque identical plastic arms (labeled as A, B, and C) with high walls at 120° angle from each other. The mouse was introduced in the center of the maze and allowed to explore the three arms freely for 8 min. Arm entry was defined as the entry of four limbs into one arm of the Y-maze. Entry into three different arms in succession (e.g., ABC, BCA, CBA, or CAB arms) was defined as one alternation. The percent alternation score was calculated by dividing the actual number of alternations by the total number of choices minus 2, expressed as a percentage as shown in the following equation:

$$ \mathrm{Alteration}\ \left(\%\right)=\left[\left(\mathrm{number}\ \mathrm{of}\ \mathrm{alterations}\right)/\left(\mathrm{total}\ \mathrm{arm}\ \mathrm{entries}-2\right)\right]\times 100\% $$

### Electrophysiology and recording

Nine-month-old APP/PS1 transgenic mice were decapitated under anesthesia with pentobarbital, and the brain was quickly removed and placed in ice-cold oxygenated artificial cerebrospinal fluid (aCSF) comprised of 110 mM NaCl, 2.5 mM KCl, 1.5 mM MgSO_4_·2H_2_O, 2.5 mM CaCl_2_, 1.25 mM NaH_2_PO_4_, 26 mM NaHCO_3_, and 10 mM d-glucose (pH 7.4). Coronal hippocampal slices (400 μm) were prepared from the resected brains of mice using a vibratome. The slices were maintained at room temperature in aCSF bubbled with 95% O_2_ and 5% CO_2_ for at least 30 min before transfer to a submersion-recording chamber, which was continually perfused with oxygenated aCSF at the rate of 1–2 mL/min. The field excitatory postsynaptic potentials (fEPSPs) in CA1 neurons were recorded by stimulating CA3 neurons. Long-term potentiation (LTP) was induced by applying high-frequency stimulation (HFS) (four 100 Hz and 1 s trains delivered 20 s apart). The LTP magnitude was quantified as the percentage change in the fEPSP slope (40%) taken during the 60-min interval after LTP induction. The electrophysiological data were acquired with an Axon multiclamp 700 B amplifier, filtered at 0.1–5 kHz, and digitized at 10 kHz, and the slope and peak amplitude of fEPSP were measured and analyzed offline using pClamp10.3 software (Molecular Devices Corp, USA). Paired-pulse facilitation (PPF) is a form of short-term synaptic plasticity, which was assessed at interstimulus intervals (ISIs) of 25, 50, 75, 100, 125, 150, and 200 ms in both groups of animals. The paired-pulse ratio was determined as the ratio between the second pulse-evoked of fEPSP and the first one.

### Golgi staining for dendritic spines

The dendritic spines were observed in the brains of 9-month-old APP/PS1 transgenic mice by Golgi-Cox staining, which were performed using the FD Rapid Golgi Stain Kit (FD Neuro Technologies, Columbia, MD, USA). The tissue sections were stained according to the manufacturer’s protocol. The images of hippocampal pyramidal neurons in CA1 and DG were viewed using Pannoramic MIDI Scanner (3DHistech Ltd., Budapest, Hungary) equipped with a GS3-U3-51S5M-C camera (FLIR, Canada), Lumencor SOLA (Beaverton, OR), and Semrock filters (Rochester, NY). The number of apical spines on hippocampal CA1 pyramidal neurons and DG neurons was counted. The second- or third-order dendritic branches were selected for quantitative analysis. The number of spines was determined per micrometer of dendritic length from 10 photographs per mouse in the digitized images. We calculated the spine density indicated by the number of spines per 10-μm branches.

### Microdialysis and ELISA

In vivo microdialysis was carried out according to our previous report [29]. Microdialysis enables direct assessment of interstitial fluid (ISF) Aβ in the setting of intact, complex neural networks [9]. The parameters for the microdialysis probes used for in vivo experiments were as follows: 220-μm OD membrane is made of hydrophilic cellulose and does not absorb Aβ (MBR-1-5 brain microdialysis probe: length of the membrane was 1 mm, 35 kDa molecular weight cut-off; guide cannula: length of the cannula was 5 mm; linear microdialysis probes; bioanalytical systems, West Lafayette, USA). Guide implantation surgery was performed [30, 31]. Briefly, a separate group of 9-month-old AD mice were anesthetized with isoflurane and fixed in a stereotactic frame (SR-5 M, Narishige, Tokyo, Japan). The two holes were made above the hippocampus (coordinates, bregma: AP: − 2.18 mm, ML ± 2.3 mm, and DV − 2.1 mm) and perforant path (coordinates, bregma: AP − 4.24 mm, ML ± 2.9 mm, and DV − 1.6 mm). To assess ISF Aβ in the hippocampus and perforant path of awake mice, in vivo microdialysis was performed. MBR-5 guide cannulas were stereotactically inserted into the brain and cemented using dental cement. The mice were kept awake during microdialysis. The constant flow rate was 1 μL/min. Microdialysis samples were collected hourly using a refrigerated fraction collector. The concentration of Aβ_1–42_ extracts in the microdialysis fractions were measured with ELISA (KHB3544, Thermo).

### Western blot

Hippocampal tissues from 9-month-old APP/PS1 transgenic mice were homogenized in radioimmunoprecipitation assay buffer (RIPA buffer) and protease inhibitor cocktail. Also, cells obtained from different treatments were lysed in RIPA buffer containing a protease inhibitor cocktail. The following antibodies were used: anti-APP (1:1000, Sigma), anti-NgR (1:1000, Millipore), anti-sAPPα (1:100, IBL), anti-sAPPβ (1:500, Covance), anti-β-site APP cleaving enzyme 1 (BACE1) (1:1000, abcam), anti-β-CTF and anti-α-CTF (1:1000, Sigma), anti-ionized calcium-binding adapter molecule 1 (Iba1) (1:1000, WAKO), glial fibrillary acidic protein (GFAP) (1:1000, DAKO) or anti-β actin antibody (1:3000, Abcam), anti-RhoA (1:1000, Sigma), and anti-ROCK2 (1:1000, Sigma). The membranes were incubated with secondary goat anti-rabbit and mouse IgG (1:5000, Thermo) and electrochemiluminescence (ECL, Millipore) reagent. The band signals were detected using BIO-RAD (Hercules, CA, USA) gel analysis software.

### Immunohistochemistry

Following the brains of APP/PS1 transgenic mice, serial 10-μm coronal sections were made with a cryostat (Leica CM 1850, Leica Microsystems AG, Wetzlar, Germany). Five mice per group were analyzed, and nine hippocampal sections from each APP/PS1 transgenic mouse were selected for immunohistochemical staining. The sections were stained with 0.05% 3, 3′-diaminobenzidine (DAB). Whole images were captured using a Pannoramic MIDI (3D Histech, Hungary) equipped with a GS3-U3-51S5M-C camera (FLIR, Canada), Lumencor SOLA (Beaverton, OR), and Semrock filters (Rochester, NY). The Aβ plaque load was quantified by the areas of Aβ plaques in the cortex and hippocampus divided by the total area of the corresponding sites. The microglia- and astrocyte-positive area were quantified as the GFAP^+^ or Iba1^+^ area divided by the total area of the hippocampus. Image-Pro Plus 6.0 image analysis software (Media Cybernetics, MD, USA) was used to analyze the images.

### Cell cultures, transfection, and treatments

HEK293 cells stably expressing human APP695 Swedish mutant (APPswe/HEK293 cells) were grown in Dulbecco’s modified Eagle’s (DMSO) medium supplemented with 10% fetal bovine serum and maintained in medium containing 400 μg/mL G418. According to the gene sequence of mouse NgR, a small interfering RNA (siRNA) targets NgR. The siRNA was designed and synthesized by Sigma (Shanghai, China). The sequences were as follows:

NgR siRNA, sense: 5′-UUCUCCGAACGUGUCACGUTT-3′

antisense: 5′-ACGUGACACGUUCGGAGAATT-3′

Control siRNA, sense: 5′-GCCGAAAUCUCACUAUCCUTT-3′

antisense: 5′-AGGAUAGUGAGAUUUCGGCTT-3′

Plasmids carrying specific NgR gene shRNA and control shRNA were constructed by Obio Technology, Shanghai, China. The sequences were as follows:

NgR shRNA, sense: 5′-GATCCCCGCCGAAATCTCACTATCCTTTCAAGAGA AGGATAGTGAGATTTCGGCTTTTT-3′

control shRNA, sense: 5′-GATCCCCTTCTCCGAACGTGTCACGTTTCAAGAGA ACGTGACACGTTCGGAGAATTTTTTGTAC-3′

The overexpression of NgR human NgR-coding DNA fragments were generated and inserted into pCDNA-CMV vector (GenePharma, Shanghai, China). APPswe/HEK293 cells and neuroblastoma N2a cells were harvested 48 h post-transduction. For transfections, equivalent amounts of cells were plated, and transfections were performed using Lipofectamine 3000 (Invitrogen, USA) according to the manufacturer’s instructions. For Rho inhibition, Y-27632 (Selleck, USA) and Fasudil (Selleck, USA) were dissolved in H_2_O. Forty-eight-hour post-transduced cells were treated with drugs at 50 μM for 10 h.

### Reverse transcriptase-PCR (RT-PCR)

Total RNA was extracted from hippocampal tissue and cells with the TRIzol reagent according to the manufacturer’s instructions. Primed RNA (1 μg) was reverse-transcribed with TransScript One-Step gDNA Removal and cDNA Synthesis Super Mix (TransGen Biotech, Beijing, China). The primers for NgR, APP, and the housekeeping gene GAPDH (Life Technologies, Thermo Fisher Scientific-CN, Shanghai, China) are listed in Table 1. The thermocycling conditions for real-time PCR were 95 °C for 2 min, followed by 30 cycles of 94 °C for 45 s, 60 °C for 45 s, and 72 °C for 60 s. Following 30 cycles, elongation was performed at 72 °C for 5 min. The PCR products were analyzed by densitometry using BIO-RAD (Hercules, CA, USA) gel analysis software.

**Table 1:** Primer sequences for RT-PCR analysis

Target mRNA sequences	Primer sequence
APP (human)	5′-GGCGGAGCAGACACAGACTA-3′
	5′-ACCTCATCACCATCCTCATCGT-3′
NgR (human)	5′-ATGCTACAATGAGCCCAAGG-3′
	5′-GAGCTGTGCATTATCGCTGA-3′
GAPDH (human)	5′-GCACCGTCAAGGCTGAGAAC-3′
	5′-TGGTGAAGACGCCAGTGGA-3′
APP (mouse)	5′-GGCCCTCGAGAATTACATCA-3′
	5′-GTTCATGCGCTCGTAGATCA-3′
GAPDH (mouse)	5′-TCACCACCATGGAGAAGGC-3′
	5′-GCTAAGCAGTTGGTGGTGCA-3′

### Immunofluorescence

APPswe/HEK293 cells were coated coverslips in 24-well culture dishes before transfection. After transfection, cells were incubated with primary antibodies including rabbit anti-APP (1:100, Sigma), mouse anti-EEA1 (1:100, cell signaling technology), mouse anti-Rab7 (1:50, abcam), and mouse anti-LAMP1 (1:10, abcam). The images were captured using a Pannoramic MIDI (3D Histech, Hungary) equipped with a GS3-U3-51S5M-C camera (FLIR, Canada), Lumencor SOLA (Beaverton, OR), and Semrock filters (Rochester, NY). Pearson’s coefficients of colocalization were analyzed by ImageJ (NIH).

### Statistical analysis

All values were reported as mean ± SEM. Simple comparisons between two groups were analyzed using the Student 2-tailed unpaired *t* test. Multiple comparisons between the groups were performed using 2-way ANOVA followed by post hoc analysis on SPSS 20.0 software. The escape latency in the Morris water maze was analyzed by two-way repeated measures ANOVA. Prism software (GraphPad Software) was used for all the graphs. A value of *P* < 0.05 was considered statistically significant.

## Results

### Knockdown of NgR in the perforant path attenuates cognitive deficits in APP/PS1 transgenic mice

Cognitive deficits are the early component of AD. In order to examine whether the perforant path NgR impacts learning and memory, we knocked down NgR of the perforant path by stereotactic injecting AAV into the perforant path of 6-month-old APP/PS1 transgenic mice. Three months after the injection, GFP labeling was detected by fluorescence imaging to confirm the location of injection (Additional file 1: Figure S1 A-B). Western blotting was used to confirm a strong reduction of NgR in the perforant path by AAV-shNgR injection (Additional file 1: Figure S1 C-D). Animals were subjected to a series of behavioral tests including Morris water maze, passive avoidance, and Y-maze.

**Fig. 1:**
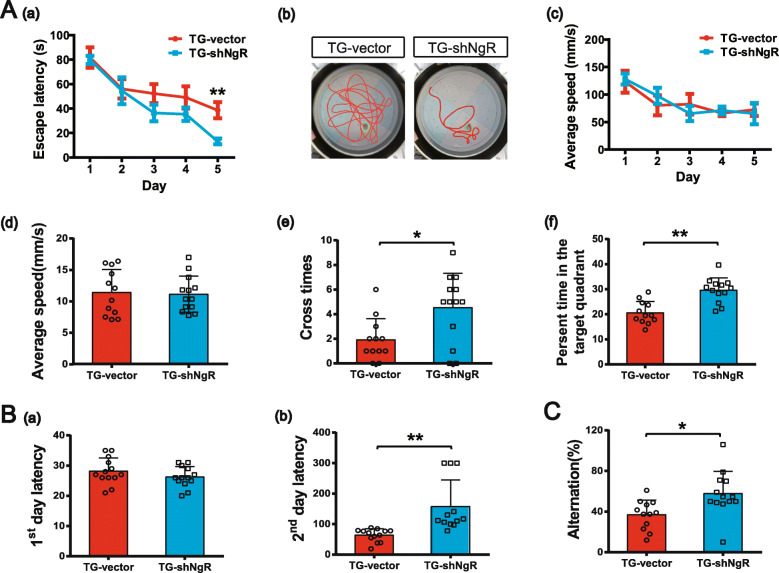
Knockdown of NgR in the perforant path attenuates cognitive impairment in APP/PS1 transgenic mice. TG-vector: AAV-vector APP/PS1 transgenic mice. TG-shNgR: AAV-shNgR APP/PS1 transgenic mice. **A** Mice were subjected to the Morris water maze test, **B** the passive avoidance test, and the **C** Y-maze spontaneous alteration test. **A** (a) The mean escape latency. **A** (b) Tracings of the typical swim patterns. **A** (c, d) The swimming speed during the acquisition training and the test phase. **A** (e) The number of crossing of the platform area. **A** (f) The percentage of the time spent by the mice in the target quadrant. **B** (a, b) The latency of APP/PS1 transgenic mice during the passive avoidance test. **C** The alternation score of the Y-maze spontaneous alteration test. Data are presented as mean ± SEM. *n* = 13 male mice/group. Data in **A** (a) and (c) were analyzed by repeated measures ANOVA with post hoc test, **P < 0.01. Data in **A** (d, f), **B**, and **C** were analyzed by Student’s *t* test, TG-shNgR mice vs TG-vector mice. *P < 0.05; **P < 0.01

The Morris water maze test was performed to examine the effect of knockdown of NgR on spatial working memory. The analysis of variance for repeated measures indicated a significant effect in the acquisition phase of learning (a latency to find the platform) [*F*_(1, 20)_ = 106.095, *p* < 0.001]. The AAV-shNgR APP/PS1 transgenic (TG-shNgR) mice exhibited improved learning in the task of locating the escape platform, which was indicated by the decreased escape latencies during the trials on the 5th day compared to the control (TG-vector) mice; the TG-shNgR mice exhibited shorter escape latency (Fig. 1A (a)). The repeated measures ANOVA showed a significant difference between-subjects effects [*F*_(1, 20)_ = 4.446, *p* = 0.048] and no significant difference within-subjects effects (time × group) [*F*_(4, 80)_ = 2.047, *p* = 0.096]. Representative swimming traces of mice are shown in Fig. 1A (b). No significant difference was shown in the swimming speed between the two groups (Fig. 1A (c, d)), which could eliminate the interference of swimming speed to the escape latencies [32]. In the probe trial, TG-shNgR mice showed improved memory retention by recording significantly increased number of platform crossing than TG-vector mice (Fig. 1A (e)). The proportion of time spent in the target quadrant by TG-shNgR mice was significantly higher than that by TG-vector mice (Fig. 1A (f)). These results demonstrated that the downregulation of NgR ameliorated the impaired learning and memory function in the APP/PS1 transgenic mice.

The passive avoidance test was used to detect the emotional learning and memory. In the acquisition session (training; day 1), no significant differences in the latency to enter the dark compartment were observed between the two groups (Fig. 1B (a)). Meanwhile, the latency of TG-shNgR mice to the dark compartment was prolonged compared to the TG-vector mice on the second day (Fig. 1B (b)), suggesting that downregulation of NgR significantly improved the emotional learning and memory in the APP/PS1 transgenic mice.

In the Y-maze test for short-term spatial memory, the percentage of alternation was significantly increased in TG-shNgR mice compare with TG-vector mice even though the total number of entries was not affected significantly (Fig. 1C). Taken together, the NgR reduction could improve the short-term and long-term memory functions and rescue the cognitive deficits in APP/PS1 transgenic mice.

### NgR reduction improves synaptic plasticity in APP/PS1 transgenic mice

**Fig. 2:**
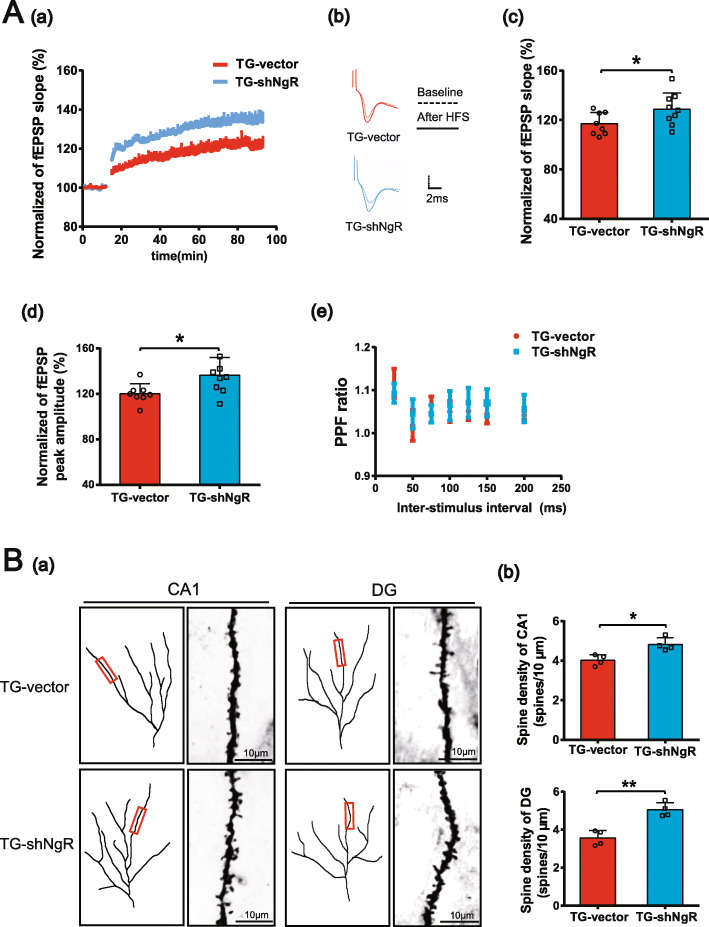
NgR reduction improves synaptic plasticity in APP/PS1 transgenic mice. **A** (a) The effects of HFS on the fEPSP initial slope. **A** (b) Representative fEPSP traces for data shown. **A** (c) Cumulative data showing the mean fEPSP slope 60 min post-HFS. **A** (d) Cumulative data showing the mean fEPSP peak amplitude 60 min post-HFS. **A** (e) Cumulative data showing the PPF ratio. **B** (a) Analysis of the density of spines of each group of mice at 9 months of age. **B** (b) The density of spines in the hippocampus of each group of the mice was analyzed. Scale bars 10 μm. Data are presented as mean ± SEM. *n* = 4 male mice/group. The statistical analysis was performed by Student’s *t* test. *P < 0.05; **P < 0.01

Cognitive impairment is strongly associated with synaptic loss and synaptic functional abnormalities induced by Aβ deposit in AD [33]. Thus, we investigated whether the downregulation of NgR could prevent impairments in synaptic plasticity in the hippocampus. LTP is widely considered to be one of the major cellular mechanisms of learning and memory [34]. In our study, it was induced by HFS in the CA1 region of 9-month-old TG-shNgR mice and TG-vector mice (Fig. 2A (a, b)). TG-vector mice showed a lower LTP compared with TG-shNgR mice indicated by a significant reduction in the fEPSP slope (Fig. 2A (c)) and fEPSP peak amplitude (Fig. 2A (d)). At the same time, the PPF in the hippocampal CA1 region was measured immediately. There was no significant difference between the two groups (Fig. 2A (e)). These results demonstrated that reducing NgR expression in the perforant path by AAV-shNgR improves LTP of APP/PS1 transgenic mice. Loss of dendritic spines in the hippocampus contributes to LTP and cognition impairment in APP/PS1 transgenic mice [33]. We therefore studied whether NgR knockdown might improve spine density in the brains of APP/PS1 transgenic mice using Golgi staining. The TG-shNgR mice showed significant increases in the density of spines in CA1 and DG compared with TG-vector mice (Fig. 2B (a, b)). The results indicated that decreasing the expression of NgR could prevent the loss of spines of APP/PS1 transgenic mice. All these results indicate that the downregulation of NgR improves synaptic plasticity in APP/PS1 transgenic mice.

### Downregulation of NgR reduces Aβ deposition

**Fig. 3:**
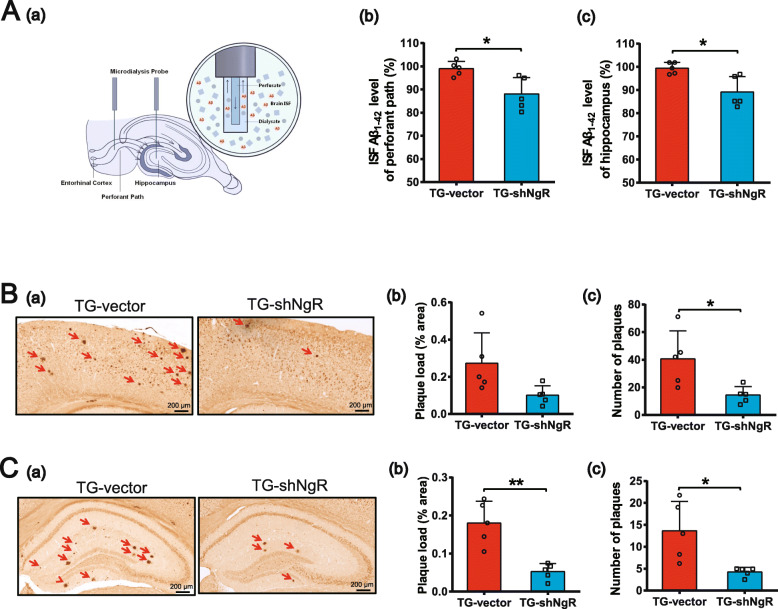
Downregulation of NgR reduces Aβ generation in APP/PS1 transgenic mice. **A** (a) Microdialysis experiments detected soluble Aβ in the brain. **A** (b, c) ISF Aβ_1–42_ levels were monitored in the perforant path and hippocampus. **B** (a) The coronal sections of the cortex were stained with an antibody against Aβ. **B** (b) The size of Aβ plaques was quantified and expressed as the percentage of areas occupied by Aβ plaques in the cortex. **B** (c) The numbers of Aβ plaques in the cortex were quantified and expressed as the amount of Aβ plaques per square millimeter. **C** (a–c) The size and numbers of Aβ plaques in the hippocampus were quantified. Scale bars 200 μm. Data are presented as mean ± SEM. *n* = 5 male mice/group. The statistical analysis was performed by Student’s *t* test. *P < 0.05; **P < 0.01

Based on the results that NgR reduction could rescue cognitive deficits, which has been identified as a major consequence of increased Aβ production, we reasoned that changes in the NgR of the perforant path may have an effect on Aβ generation. Soluble Aβ in ISF has been shown to reflect total soluble Aβ in extracellular pools and is significantly correlated with the amyloid deposition of the brain [30, 35]. Microdialysis has contributed a very important technology to the evaluation of soluble Aβ in AD [30, 31, 36, 37]. We used microdialysis to assess ISF Aβ_1–42_ metabolism both in the perforant path and hippocampus of APP/PS1 transgenic mice (Fig. 3A (a)). The ISF Aβ concentrations in the perforant path (Fig. 3A (b)) and hippocampus (Fig. 3A (c)) of TG-shNgR mice decreased in comparison with TG-vector mice. In addition to soluble Aβ analysis, coronal sections of the cortex and hippocampus were stained with an antibody against Aβ in APP/PS1 transgenic mice (Fig. 3B (a), C (a)). Quantification analysis showed that the size (Fig. 3B (b) and C (b)) and numbers (Fig. 3B (c) and C (c)) of Aβ plaques were significantly reduced in the cortex and hippocampus of TG-shNgR mice compared with TG-vector mice. These results suggest an inhibitory effect of reducing NgR in the perforant path on the generation and accumulation of Aβ plaques in APP/PS1 transgenic mice.

### NgR reduction suppresses the activation of microglia and astrocytes in APP/PS1 transgenic mice

**Fig. 4:**
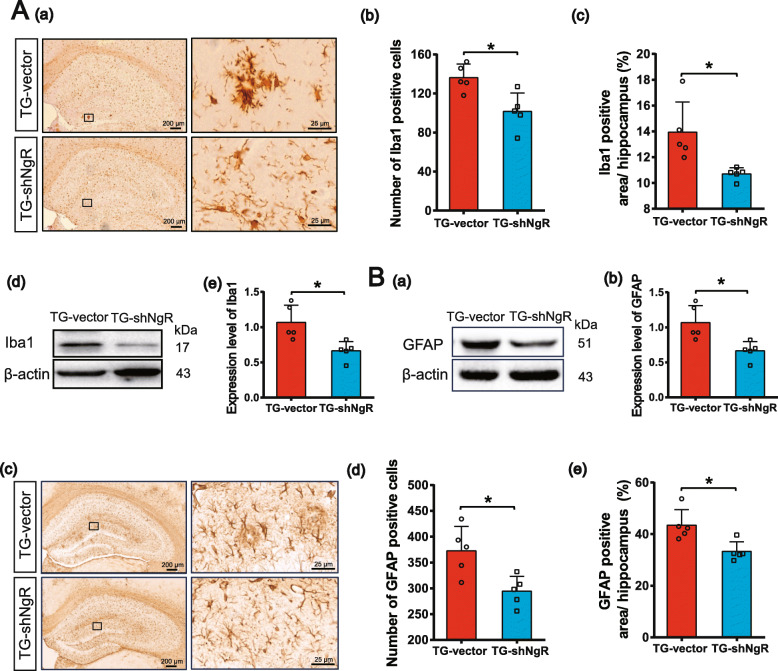
NgR reduction decreases activation of astrocytes and microglia in the hippocampus. **A** (a) Mouse brain sections were processed for anti-Iba1 immunohistochemistry as indicated. **A** (b, c) The numbers and percentage of the Iba1-positive area in the hippocampus. **A** (d, e) Representative bands and analysis of the Iba1 protein level. **B** (a, b) Representative bands of and analysis of the GFAP protein level. **B** (c) Mouse brain sections were processed for anti-GFAP immunohistochemistry as indicated. **B** (d, e) The numbers and percentage of the GFAP-positive area in the hippocampus. Data are presented as mean ± SEM. *n* = 5 male mice/group. The statistical analysis was performed by Student’s *t* test. *P < 0.05

Astrocyte and microglia activation is associated with amyloid plaque formation, neuronal and synaptic dysfunction, cell death, and further neurodegeneration [38, 39]. Neuroinflammation has been recognized as an important cause of AD and is associated with disease severity. Thus, we addressed whether NgR knockdown would inhibit glial activation in APP/PS1 transgenic mice. We performed immunohistochemical staining on coronal sections of the hippocampus with antibodies against Iba-1 and GFAP (markers for microglia and astrocyte respectively) (Fig. 4A (a), B (c)). The results showed that the positive cells and area percentage of both microglia and astrocytes in the hippocampus of TG-shNgR mice decreased compared with TG-vector mice (Fig. 4A (b, c), B (d, e)). Western blot analysis also confirmed the above results (Fig. 4A (d, e), B (a, b)). These results suggest that downregulation of NgR can attenuate glial activation induced by amyloid plaques in APP/PS1 transgenic mice.

### NgR affects APP processing

**Fig. 5:**
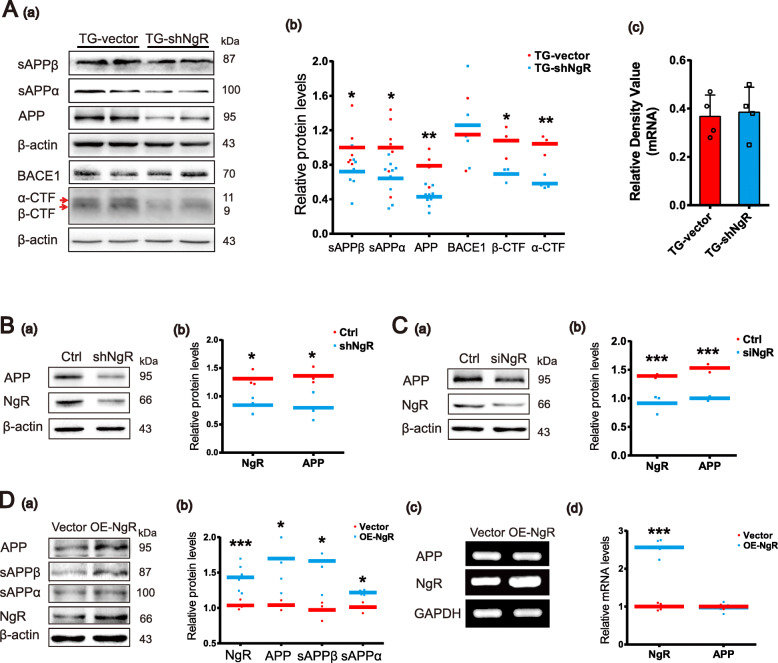
NgR changes APP and its proteolytic amyloidogenic fragment levels in vivo and in vitro. **A** (a) Representative images and Western blot analysis of expression levels of sAPPβ, sAPPα, APP, BACE1, β-CTF, or α-CTF in the hippocampus. **A** (b) Densitometry analysis of protein levels of APP and its proteolytic amyloidogenic fragments. **A** (c) PCR analysis showing the mRNA expression level of APP in the hippocampus. *n* = 3–9 male mice/group. **B** (a, b) Neuroblastoma N2a cells were transfected with plasmids, densitometry analysis of protein levels of APP. **C** (a, b) Representative bands of the Western blot and densitometry analysis of protein levels of APP by siRNA interference in APPswe/HEK293 cells. **D** (a, b) Overexpression of NgR by transfecting with plasmids in APPswe/HEK293 cells, densitometry analysis of protein levels of APP and its proteolytic amyloidogenic fragments. **D** (c, d) Representative micrographs of PCR and densitometry analysis of APP. Data are presented as mean ± SEM. *n* = 3–6. The statistical analysis was performed by Student’s *t* test. *P < 0.05; **P < 0.01; ***P < 0.001

There are both amyloidogenic and non-amyloidogenic processing of APP, and decreased Aβ could be the result of increased α-secretase cleavage and decreased β-secretase cleavage. We quantified the products of α- and β-secretases’ cleavage, a large α- and β-fragment ectodomain of APP (sAPPα and sAPPβ), and the corresponding small cytoplasmic fragments, α-CTF and β-CTF, in order to understand why NgR knockdown decreases Aβ level. Both sAPPα/α-CTF and sAPPβ/β-CTF were decreased significantly in the hippocampus in TG-shNgR mice, compared with TG-vector mice (Fig. 5A (a, b)). BACE1 protein expression level showed no significant difference between the two groups. Additionally, APP protein levels of TG-shNgR mice were substantially less than those of TG-vector mice (Fig. 5A (a, b)) and APP mRNA levels remained unaffected by NgR reduction (Fig. 5A (c)).

Next, we performed the in vitro experiments to further confirm the above results. We knocked down NgR effectively using shRNA constructs in neuroblastoma N2a cells (Fig. 5B (a)). Densitometry analysis indicated that APP protein was obviously reduced following RNAi downregulation of NgR (Fig. 5B (b)). In APPswe/HEK293 cells that express mutant APP, NgR knockdown by siRNA interference also significantly decreased the protein level of APP (Fig. 5C (a, b)). Overexpression of NgR in APPswe/HEK293 cells significantly increased the APP protein level as well as the sAPPα and sAPPβ production (Fig. 5D (a, b)), while the mRNA levels of APP did not change significantly by overexpression of NgR (Fig. 5D (c, d)).

Both in vivo and in vitro results showed that NgR had the same effect on the total APP protein level and α/β-secretase cleavage, suggesting NgR might interfere with the intracellular process that reduces APP supply for both the amyloidogenic and non-amyloidogenic pathways.

### NgR reduction promotes APP traffic to lysosomes by Rho/ROCK pathway

**Fig. 6:**
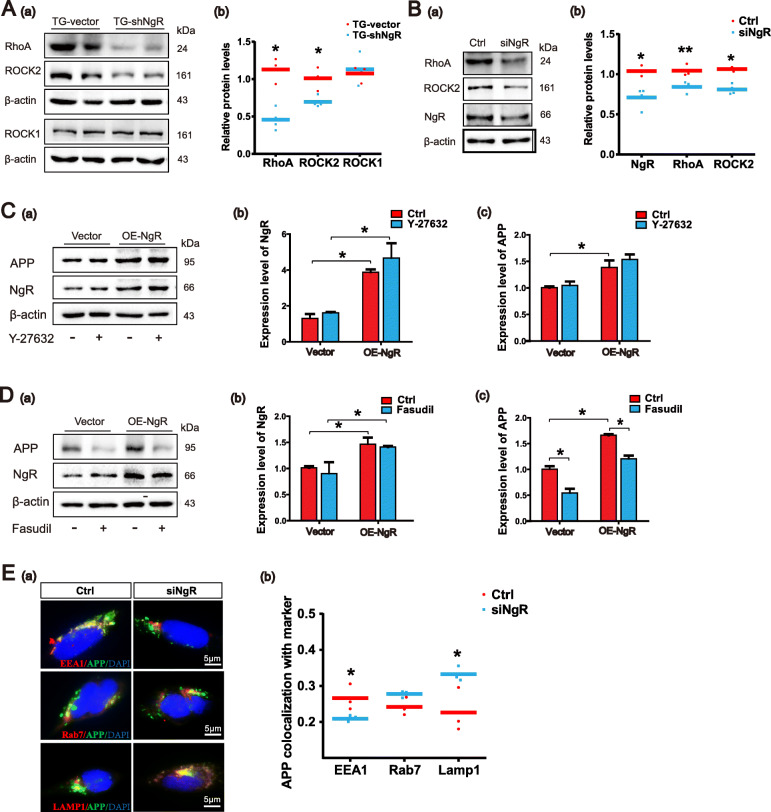
NgR reduction promotes APP trafficking to lysosomes by Rho/ROCK2 pathway. **A** (a, b) Representative bands of the Western blot and densitometry analysis of RhoA, ROCK1, and ROCK2 levels in APP/PS1 transgenic mice. *n* = 3–4 male mice/group. **B** (a, b) Western blot analysis showing the expression levels of RhoA and ROCK2 following siRNA-induced downregulation of NgR in APPswe/HEK293 cells. **C**, **D** APPswe/HEK293 cells were transduced with plasmid to overexpress NgR and exposed to Y-27632 (50 μM) and Fasudil (50 μM) for 10 h. **C** (a–c) Representative bands of the Western blot and densitometry analysis of NgR and APP levels by incubating Y-27632. **D** (a–c) Representative bands of the Western blot and densitometry analysis of NgR and APP levels by incubating Fasudil. **E** (a) APP colocalization with the following organelle markers: EEA1, Rab7, and LAMP1 were evaluated after knocking down NgR in APPswe/HEK293 cells. Representative images of APP (green) and organelle markers (red), with colocalization (yellow), are shown in the merged and zoom-in image. **E** (b) Quantitative analyses of APP and organelle marker staining. Scale bars 5 μm. Data are presented as mean ± SEM. *n* = 3–4. The statistical analysis was performed by Student’s *t* test and 2-way ANOVA. *P < 0.05; **P < 0.01

Intracellular APP trafficking is critical for APP localization and processing. NgR, the receptor of myelin inhibitors, increases the GTP bound state of the neuronal cytoskeleton regulatory factor RhoA, to activate the RhoA/ROCK pathway [40]. And it is reported that inhibition of ROCK2 activity may reduce the amyloidogenic process from APP [2]. Our result showed that the levels of RhoA and ROCK2, but not ROCK1 of TG-shNgR mice were significantly decreased (Fig. 6A (a, b)). Furthermore, the in vitro experiment demonstrated that the RhoA and ROCK2 levels in siNgR-treated APPswe/HEK293 cells were significantly decreased (Fig. 6B (a, b)).

Therefore, we hypothesized that inhibiting ROCK may result in the reduction of APP. To determine this speculation, APPswe/HEK293 cells were treated with Y-27632 (ROCK1 antagonist) and Fasudil (ROCK2 antagonist) respectively and the protein levels of APP were examined by Western blot assay. The quantitative results showed that Fasudil remarkably decreased the APP level (Additional file 2: Figure S2). Subsequently, APPswe/HEK293 cells with overexpression of NgR were treated with Y-27632 and Fasudil respectively and the protein levels of APP and NgR were examined by Western blot assay (Fig. 6C (a), D (a)). The quantitative results showed that inhibiting the activity of ROCK2 kinase, not ROCK1 kinase, could reverse the elevation of APP protein induced by overexpression of NgR (Fig. 6C (b, c), D (b, c)). As RhoA/ROCK2 pathway is involved in APP degradation in lysosome [2], so we examined whether NgR modulates APP transport to lysosome. NgR was reduced in APPswe/HEK293 cells growing on coverslips. After 48 h, the cells were stained for APP and markers for early endosomes (EEA1), late endosomes (Rab7), or lysosomes (LAMP1) (Fig. 6E (a)). Confocal microscopy observation showed that APP colocalization with EEA1 was reduced whereas APP colocalization with LAMP1 was markedly increased by NgR reduction (Fig. 6E (b)). APP colocalization with Rab7-positive compartments also appeared elevated by NgR silence although the difference was not statistically significant (Fig. 6E (b)). These observations suggest that NgR reduction decreases the APP presence in early endosomes and promotes APP traffic to lysosomes by Rho/ROCK2 pathway.

## Discussion

The exact mechanisms of APP contributing to the development of dementia are unknown by far, but cleavage of APP by secretases leading to the formation of a smaller peptide, Aβ, is considered a crucial step. Deposition of Aβ is widely regarded as a marker of the pathology of AD and is associated with other pathological changes such as the brain cell death, accumulation of amyloid plaques, and appearance of neurofibrillary tangles. In AD patients, the EC, origination of the perforant path, is one of the most severely affected areas, particularly at earlier stages of the disease [4]. Moreover, electrical activity within the pathway modulates interstitial fluid Aβ levels, which can be blocked by tetrodotoxin (TTX), a specific sodium channel blocker [9]. NORs are the naked regions of the axolemma and intermittently distribute along myelinated axons, implying potential interactions between exposed axonal molecules and the extracellular partners. According to our previous observation that APP is specifically located at NORs in the central nervous system [10], we propose that the NORs, to some extent, are important Aβ releasing sites, which correlate with its deposition along the perforant path at the onset of AD.

NgR is an important molecule at NORs, which participate in limiting injury-induced axonal growth and experience-dependent plasticity in the adult brain [12, 13]. In this role, it serves as a receptor for three myelin inhibitor proteins, Nogo, MAG, and OMgp, signaling to activate Rho GTPase in axons [13, 40]. Recently, it is reported that NgR is involved in AD pathological features, but the results have been conflicting and the detailed mechanism needs further investigation. In the current study, NgR knockdown in the perforant path was shown to ameliorate the cognitive and synaptic deficits in APP/PS1 transgenic mice (Figs. 1 and 2), which could be attributed, at least in part, to the reduced level of soluble and deposited Aβ (Fig. 3) and related weakened neuroinflammation (Fig. 4). Because Aβ is generated and released from neuronal terminals into the extracellular space, activity-related changes in Aβ levels would likely occur and are able to detect immediately in brain interstitial fluid. Microdialysis enables direct and frequent assessment of ISF Aβ concentrations in the setting of intact, complex neural networks. To determine whether knockdown NgR in the perforant path influences ISF Aβ levels in vivo, microdialysis probes were inserted into the hippocampus and perforant path separately to measure ISF Aβ levels from the awake APP/PS1 transgenic mice. A sensitive ELISA for Aβ_1–42_ was used to assess ISF Aβ. We found that ISF Aβ concentrations both in the perforant path (Fig. 3A (b)) and hippocampus (Fig. 3A (c)) of TG-shNgR mice decreased in comparison with TG-vector mice. This data, to some extent, supports the idea that the Aβ is released, at least partially from naked region of myelinated axon-NORs. More detail evidences about the generation of Aβ in this area need to be clarified furtherly. If it is the case, reduced Aβ production in the perforant path might have functional effect directly and/or contribute to the decreased Aβ level in the hippocampus and cortex. It might be likely that NgR reduction in the perforant path lowered the Aβ level in this pathway and then reduced the “spread” of Aβ to the hippocampus and cortex, through prion-like propagation [41] or functional activity [9].

In contrast to our results, Park et al. have demonstrated that NgR knockout in APP/PS1 mice results in an increase in amyloid plaques and Aβ peptides in both the cortex and hippocampus [22]. We speculate that different manipulation on NgR gene might be the major reason that we observed results opposite to theirs. Genetic loss of NgR might affect the function of the whole brain and even the developmental processes of the APP/PS1 mice. In our study, NgR expression was selectively reduced in the perforant path as we are more interested in the role of the perforant path in the development of amyloid pathology.

Neuroinflammation characterized by astrocyte and microglia activation is another common AD pathology [38, 39] that can be induced by amyloid deposition and neuronal injury. In this study, selective NgR knockdown was shown to inhibit microglia and astrocyte activation in the brain of APP/PS1 mice (Fig. 4). It is reasonable that reduced amyloid deposition and glial activation leads to improved cognitive function of APP/PS1 mice with less NgR in the perforant path, although the effect of NgR knockdown on cognitive function in wild type mice is not clear. Studies have demonstrated the role of NgR1 as a critical gate to both experience-dependent learning and anatomical plasticity [42, 43] and lacking NgR1 impairs short-term object memory [18]. However, overexpression of the NgR1 gene in forebrain neurons impairs the formation of lasting memories, suggesting impaired plasticity when NgR1 cannot be downregulated as needed [44]. Consistently, expressions of NgR are increased in patients with AD [19], where NgR knockdown could be beneficial.

Aβ reduction could be the result of decreased production and/or increased clearance of this peptide. Several studies have suggested NgR modulates Aβ levels in the brain and influences the cognitive functions of AD animals, even though the results are conflicting [21–23, 45]. It is suggested that NgR2 interferes with the interaction of APP and BACE-1 and reduces the cleavage of APP by BACE-1 [45]. Our results indicate that the BACE-1 protein level was not affected by knocking down NgR (Fig. 5A (a, b)) and downregulation of NgR decreased both α-secretase and β-secretase cleavage products as well as the total protein level of APP (Fig. 5A (a, b)). It is not known whether NgR could suppress both α-secretase and β-secretase activities; however, it is possible that NgR regulates APP processing by affecting the APP protein level without influencing the expressions and activities of the secretases. APP protein levels are affected by both gene expression and protein processing in the cells. Here, the NgR level had no effect on the mRNA level of APP, so we further investigated whether NgR played a role in APP processing.

We already know that full-length APP is synthesized in the endoplasmic reticulum (ER) and transported to the cell surface through the Golgi/trans-Golgi network (TGN), where APP can be cleaved by β-secretase and then by γ-secretase complex to produce Aβ. Alternatively, APP can be cleaved by α-secretase within the Aβ domain to release soluble APPα and preclude Aβ generation. APP can also be internalized within clathrin-coated vesicles and may be recycled back to the cell surface, retrieved back to the TGN, or processed in endosomes [46, 47]. It has been proposed that Golgi and endosomes are the major intracellular compartments of APP being processed into Aβ [48–52]. It has been reported that APP protein can be degenerated through both the proteasomal and lysosomal pathways [53] and inhibition of lysosomal or proteasomal degradation processes will cause the accumulation of APP fragments and Aβ production. Consistently, enhancing lysosomal cathepsin activity ameliorates Aβ toxicity [54, 55] and restoring the autophagy-lysosomal pathway reduced Aβ accumulation and rescued memory performance [56]. These studies suggest that APP traffic to lysosomes may reduce the APP protein level and Aβ production.

**Fig. 7:**
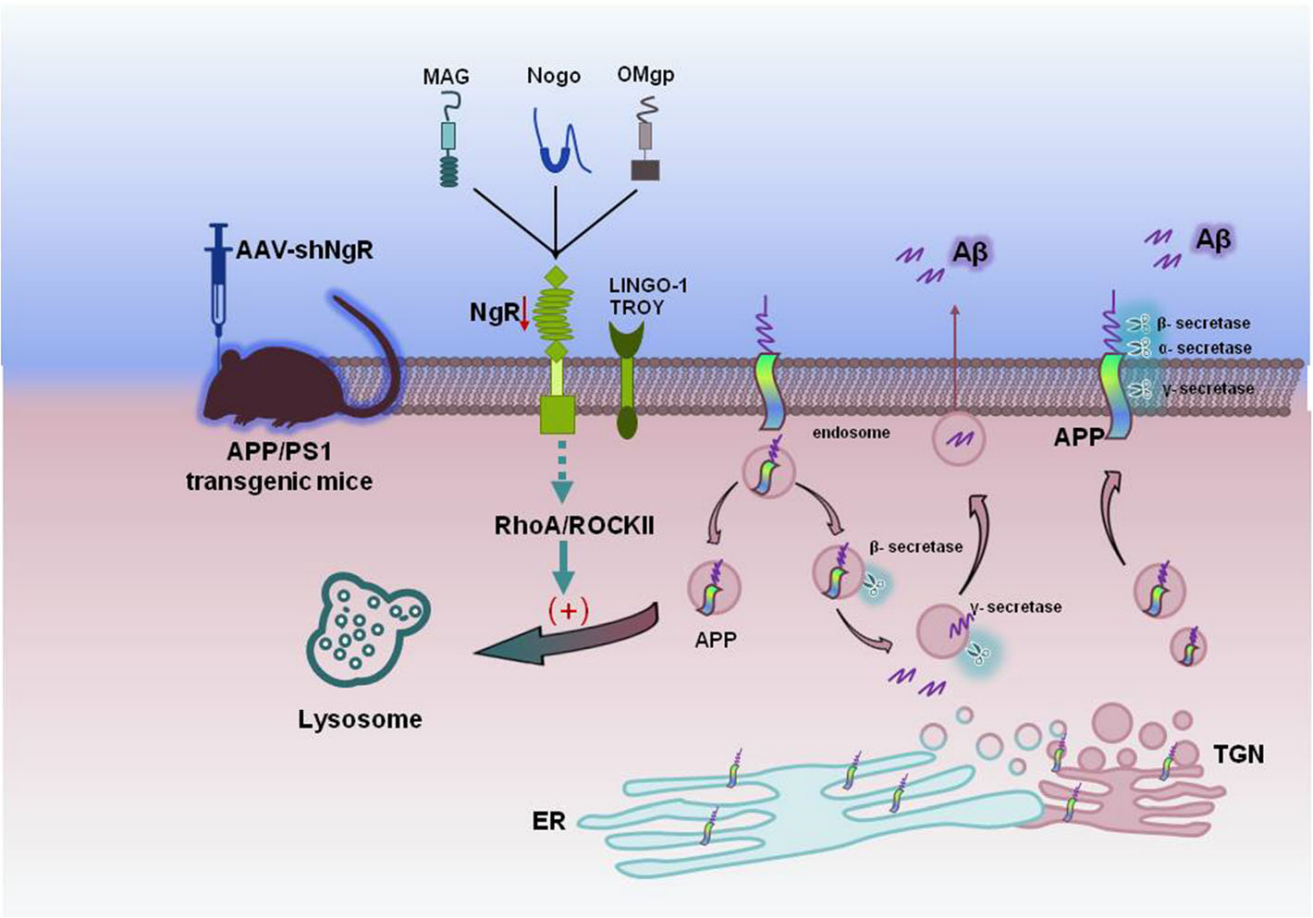
The proposed effect of NgR reduction on APP traffic to lysosome through Rho/ROCK2 pathway. Full-length APP is synthesized in the endoplasmic reticulum (ER) and transported to the cell surface or endosomes/lysosomes. Once APP is transported to the cell surface, it undergoes amyloidogenic or non-amyloidogenic processing by β-secretase or α-secretase cleavage. While partial APP is internalized and delivered to endosome, a fraction of endocytosed APP molecules is recycled back to the cell surface, and other fractions are either processed in the endosome to release Aβ or delivered to lysosome for degradation. NgR reduction suppresses the downstream Rho/ROCK2 signaling pathway which enhances APP traffic to lysosomes, thus may reduce APP distribution to other cellular compartments and decrease Aβ production

NgR is known to increase the GTP bound state of the neuronal cytoskeleton regulatory factor RhoA, a Rho GTPase family member, to activate the RhoA/ROCK pathway [40]. Notably, RhoA/ROCK2 pathway is involved in APP degradation in lysosome and inhibition of ROCK2 activity reduces amyloidogenic process from APP [2]. Our result showed that the levels of RhoA and ROCK2 are correlated with NgR levels both in APP/PS1 mice and APPswe/HEK293 cells (Fig. 6A, B). So it is possible that NgR might regulate APP protein level through Rho/ROCK pathway. Indeed, inhibition of the activity of ROCK2 kinase could reverse the elevation of APP protein induced by overexpression of NgR (Fig. 6C, D). Further, it was shown that APP localized more strongly to LAMP1-positive compartments following knockdown of NgR, suggesting that NgR suppresses APP traffic to lysosome. Together, these results implicate NgR in APP degradation and Aβ production through Rho/ROCK2 signaling pathway (Fig. 7). As a glycosylphosphatidylinositol (GPI)-anchored protein lacking intracellular component, NgR does not transduce signals directly, but instead recruits co-receptor molecules such as p75NTR or TROY [40]. However, how NgR activates Rho/ROCK2 pathway is not well understood yet. The plexin receptors regulate cell adhesion, migration, and guidance, with their intracellular region interacts directly with small GTPases of the Rho family [57, 58]. Plexins are widely expressed in neurons, performing critical functions in axon guidance and signal transduction [59]. It is of interest to investigate functional correlation between NgR and plexin receptors.

## Conclusions

In summary, our study suggests that NgR plays a critical role in APP processing through Rho/ROCK signaling pathway, and NgR knockdown in the perforant path reduces the APP level and Aβ production, which might account for the improved synaptic and cognitive function in the APP/PS1 mice. These findings provide a better understanding of the roles of the perforant path and NgR in AD pathogenesis and offer a novel therapeutic strategy and approaches for the treatment of AD.

### Supplementary information

**Additional file 1: Figure S1.** Evaluation of the infection efficiency after injection of AAV expressing plasmids pAKD-CMV-bGlobin-eGFP-H1-shNgR into the perforant path three months. Fluorescence image of AAV-mediated GFP expression at target area (**B**) and expressions of NgR in the perforant path by Western blotting (**C** Representative blot image and **D** densitometry analysis of protein levels). PP: perforant path. Scale bars: 500 μm. The statistical analysis was performed by Student′s *t*-test. *P < 0.05.

**Additional file 2: Figure S2.** APP expression levels in APPswe/HEK293 cells treated with different concentration of Y-27632 or Fasudil for 10 h. **A** Representative blot image and **B** densitometry analysis of APP levels after incubating Y-27632. **C** Representative blot image and **D** densitometry analysis of APP levels after incubating Fasudil. The statistical analysis was performed by one-way ANOVA. *P < 0.05; **P < 0.01.

## Data Availability

All data generated in this study are available from the corresponding author on reasonable request.

## Abbreviations

NgR: Nogo receptor
Aβ: Amyloid beta
AD: Alzheimer’s disease
AAV: Adeno-associated virus
ANOVA: Analysis of variance
APP: Amyloid beta precursor protein
EC: Entorhinal cortex
ROCK: Rho-associated protein kinases
BACE1: β-Site amyloid precursor protein-cleaving enzyme 1
CTFα and CTFβ: C-terminal fragments of APP
GFAP: Glial fibrillary acidic protein
Iba1: Ionized calcium-binding adaptor molecule-1
ISF: Interstitial fluid
PS1: Presenilin 1
SAPPβ: Soluble peptide APP β
shRNA: Short hairpin
NOR: Node of Ravier
MAG: Myelin-associated glycoprotein
OMgp: Oligodendrocyte myelin glycoprotein
LTP: Long-term potentiation
PPF: Paired-pulse facilitation
aCSF: Artificial cerebrospinal fluid
fEPSPs: Field excitatory postsynaptic potentials
HFS: High-frequency stimulation
siRNA: Small interfering RNA
ER: Endoplasmic reticulum
TGN: Trans-Golgi network
GPI: Glycosylphosphatidylinositol
