# Correction: Reduction of NgR in perforant path decreases amyloid-β peptide production and ameliorates synaptic and cognitive deficits in APP/PS1 mice

**DOI:** 10.1186/s13195-024-01389-9

**Published:** 2024-02-27

**Authors:** Rong Jiang, Xue-Fei Wu, Bin Wang, Rong-Xiao Guan, Lang-Man Lv, Ai-Ping Li, Lei Lei, Ye Ma, Na Li, Qi-Fa Li, Quan-Hong Ma, Jie Zhao, Shao Li

**Affiliations:** 1 Liaoning Provincial Key Laboratory of Cerebral Diseases, Department of Physiology, College of Basic Medical Sciences, Dalian Medical University, Dalian, China; 2 National-Local Joint Engineering Research Center for Drug Research and Development (R&D) of Neurodegenerative Diseases, Dalian Medical University, Dalian, China; 3 Jiangsu Key Laboratory of Neuropsychiatric Diseases, Institute of Neuroscience, Soochow University, Suzhou, China

**Correction: Alz Res Therapy 12, 47 (2020)**

**https://doi.org/10.1186/s13195-020-00616-3**

Following publication of the original article [1], the authors identified an error in Fig. 6C (a).

The corrected Fig. 6C(a) with the correct band and original data is given hereafter.

The incorrect Fig. 6:

**Fig. 6:**
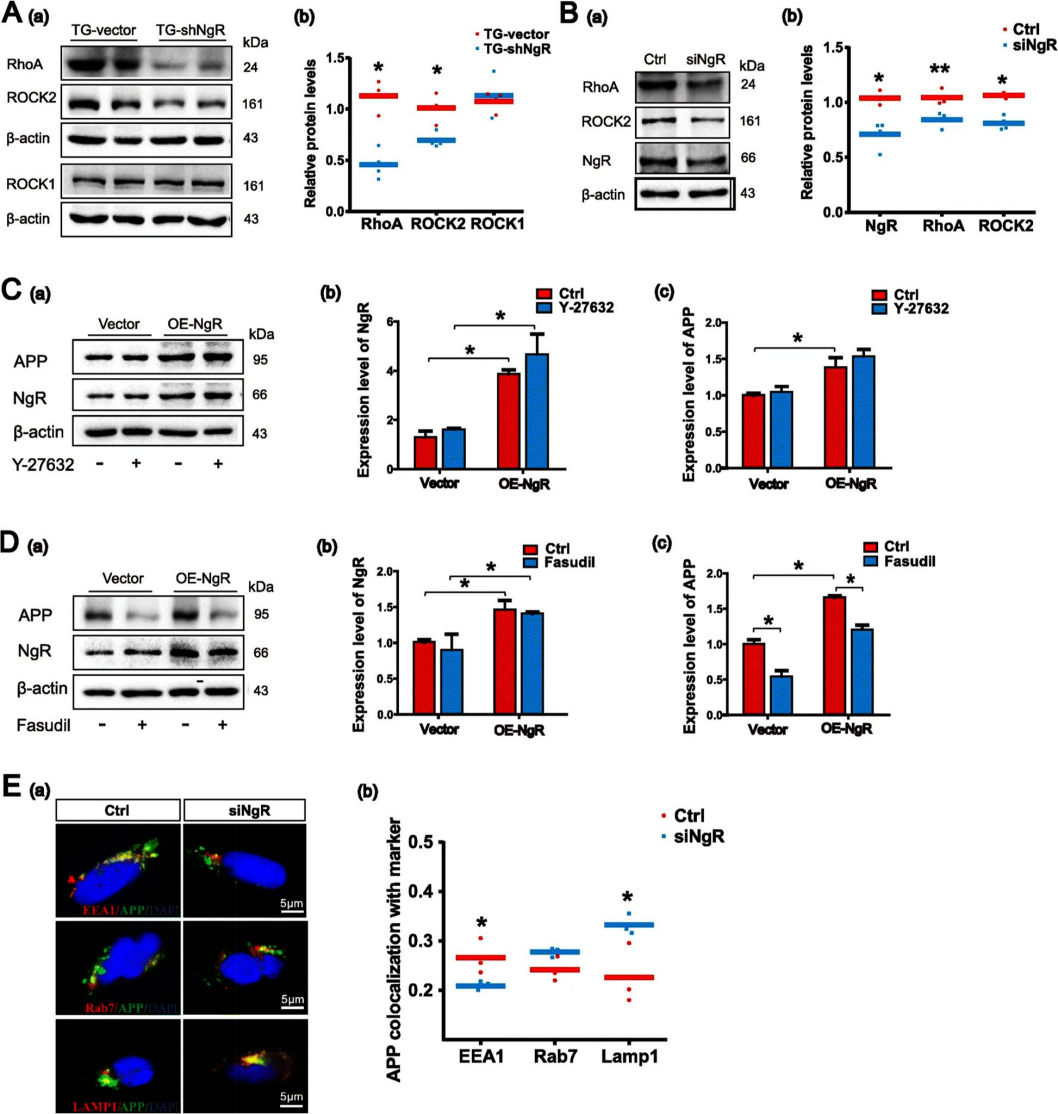
NgR reduction promotes APP trafficking to lysosomes by Rho/ROCK2 pathway. **A** (a, b) Representative bands of the Western blot and densitometry analysis of RhoA, ROCK1, and ROCK2 levels in APP/PS1 transgenic mice. *n* = 3–4 male mice/group. **B** (a, b) Western blot analysis showing the expression levels of RhoA and ROCK2 following siRNA-induced downregulation of NgR in APPswe/HEK293 cells. C, D APPswe/HEK293 cells were transduced with plasmid to overexpress NgR and exposed to Y-27632 (50 μM) and Fasudil (50 μM) for 10 h. **C** (a–c) Representative bands of the Western blot and densitometry analysis of NgR and APP levels by incubating Y-27632. **D** (a–c) Representative bands of the Western blot and densitometry analysis of NgR and APP levels by incubating Fasudil. **E** (a) APP colocalization with the following organelle markers: EEA1, Rab7, and LAMP1 were evaluated after knocking down NgR in APPswe/HEK293 cells. Representative images of APP (green) and organelle markers (red), with colocalization (yellow), are shown in the merged and zoom-in image. **E** (b) Quantitative analyses of APP and organelle marker staining. Scale bars 5 μm. Data are presented as mean ± SEM. *n* = 3–4. The statistical analysis was performed by Student’s t test and 2-way ANOVA. *P < 0.05; **P < 0.01

The correct Fig. 6:

**Fig. 6:**
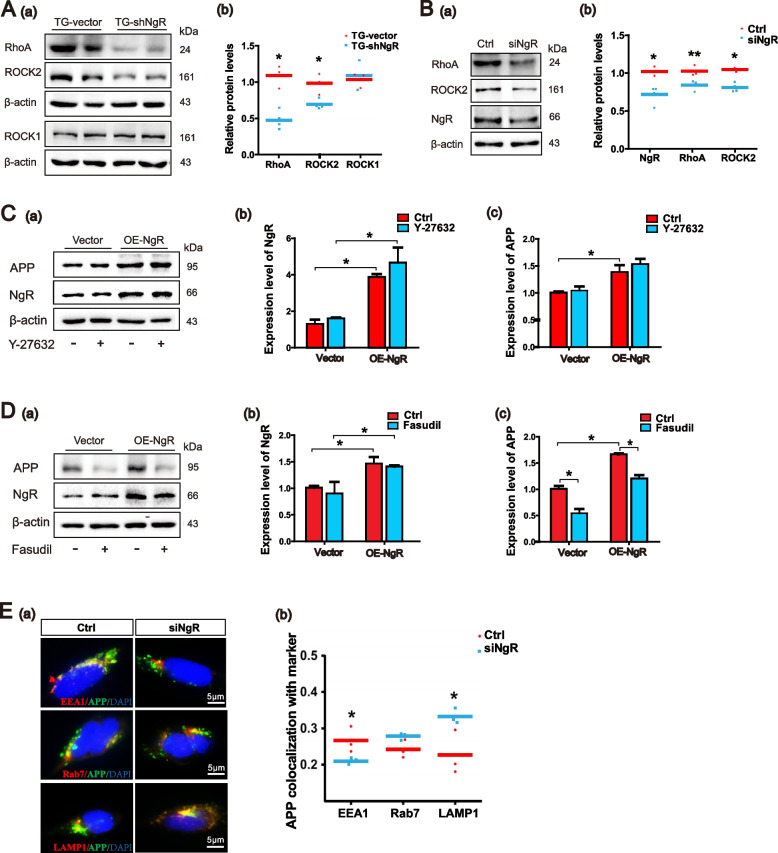
NgR reduction promotes APP trafficking to lysosomes by Rho/ROCK2 pathway. **A** (a, b) Representative bands of the Western blot and densitometry analysis of RhoA, ROCK1, and ROCK2 levels in APP/PS1 transgenic mice. *n* = 3–4 male mice/group. **B** (a, b) Western blot analysis showing the expression levels of RhoA and ROCK2 following siRNA-induced downregulation of NgR in APPswe/HEK293 cells. C, D APPswe/HEK293 cells were transduced with plasmid to overexpress NgR and exposed to Y-27632 (50 μM) and Fasudil (50 μM) for 10 h. **C** (a–c) Representative bands of the Western blot and densitometry analysis of NgR and APP levels by incubating Y-27632. **D** (a–c) Representative bands of the Western blot and densitometry analysis of NgR and APP levels by incubating Fasudil. **E** (a) APP colocalization with the following organelle markers: EEA1, Rab7, and LAMP1 were evaluated after knocking down NgR in APPswe/HEK293 cells. Representative images of APP (green) and organelle markers (red), with colocalization (yellow), are shown in the merged and zoom-in image. **E** (b) Quantitative analyses of APP and organelle marker staining. Scale bars 5 μm. Data are presented as mean ± SEM. *n* = 3–4. The statistical analysis was performed by Student’s t test and 2-way ANOVA. *P < 0.05; **P < 0.01

Figure 6C(a) has been updated above and the original article [1] has been corrected.
