# Supplementary material for: Reduction of NgR in perforant path decreases amyloid-β peptide production and ameliorates synaptic and cognitive deficits in APP/PS1 mice
Source: Alzheimers Res Ther. 2020 Apr 24;12:47. doi: 10.1186/s13195-020-00616-3 (PMC7181577; doi:10.1186/s13195-020-00616-3)
Supplement: Supplementary file 1 — Additional file 1: Figure S1. Evaluation of the infection efficiency after injection of AAV expressing plasmids pAKD-CMV-bGlobin-eGFP-H1-shNgR into the perforant path three months. Fluorescence image of AAV-mediated GFP expression at target area (B) and expressions of NgR in the perforant path by Western blotting (C Representative blot image and D densitometry analysis of protein levels). PP: perforant path. Scale bars: 500 μm. The statistical analysis was performed by Student′s t-test. *P < 0.05. [file 13195_2020_616_MOESM1_ESM.docx]

**Additional file 1**

**B**

**A**


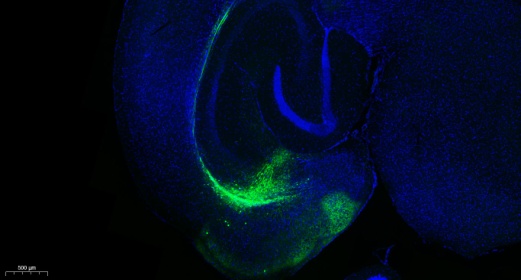

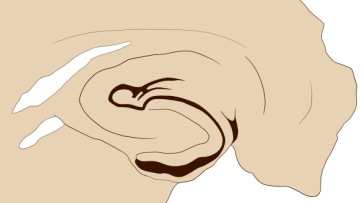


**DG**

**CA3**

**CA1**

**PP**

**500 μm**



­­­

**C**

**D**

*****

**Additional file 1: Figure S1.** Evaluation of the infection efficiency after injection of AAV expressing plasmids pAKD-CMV-bGlobin-eGFP-H1-shNgR into the perforant path three months. Fluorescence image of AAV-mediated GFP expression at target area (**B**) and expressions of NgR in the perforant path by Western blotting (**C** Representative blot image and **D** densitometry analysis of protein levels ). PP: perforant path. Scale bars: 500 μm. The statistical analysis was performed by Student′s *t*-test. **P* <0.05
