# Supplementary material for: Reduction of NgR in perforant path decreases amyloid-β peptide production and ameliorates synaptic and cognitive deficits in APP/PS1 mice
Source: Alzheimers Res Ther. 2020 Apr 24;12:47. doi: 10.1186/s13195-020-00616-3 (PMC7181577; doi:10.1186/s13195-020-00616-3)
Supplement: Supplementary file 2 — Additional file 2: Figure S2. APP expression levels in APPswe/HEK293 cells treated with different concentration of Y-27632 or Fasudil for 10 h. A Representative blot image and B densitometry analysis of APP levels after incubating Y-27632. C Representative blot image and D densitometry analysis of APP levels after incubating Fasudil. The statistical analysis was performed by one-way ANOVA. *P < 0.05; **P < 0.01. [file 13195_2020_616_MOESM2_ESM.docx]

**Additional file 2**


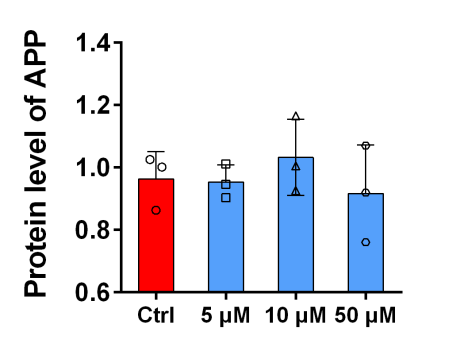


**B**

**C**

**A**





**D**

******

*****

******

**Additional file 2: Figure S2.** APP expression levels in APPswe/HEK293 cells treated with different concentration of Y-27632 or Fasudil for 10 h. **A** Representative blot image and **B** densitometry analysis of APP levels after incubating Y-27632. **C** Representative blot image and **D** densitometry analysis of APP levels after incubating Fasudil. The statistical analysis was performed by one-way ANOVA.  ******P* <0.05; *******P* <0.01
